# The Effects of Contextual Factors, Self-Efficacy and Motivation on Learners’ Adaptability to Blended Learning in College English: A Structural Equation Modeling Approach

**DOI:** 10.3389/fpsyg.2022.847342

**Published:** 2022-04-06

**Authors:** Shuhan Yang, Ruihui Pu

**Affiliations:** ^1^School of Foreign Languages and Cultures, Panzhihua University, Panzhihua, China; ^2^Rattanakosin International College of Creative Entrepreneurship, Rajamangala University of Technology Rattanakosin, Nakhon Pathom, Thailand; ^3^Faculty of Economics, Srinakharinwirot University, Bangkok, Thailand

**Keywords:** contextual factors, self-efficacy, motivation, adaptability, blended learning, College English, structural equation modeling

## Abstract

**Objective:**

Few research efforts have substantially introduced relevant studies on Chinese students’ adaptability in relation to the ineffectiveness of blended learning mode in College English. This study is guided by social cognitive theory, and related literature has been reviewed concerning adaptability. In this study, we aim to examine the involved relationships among contextual factors, self-efficacy, motivation, and adaptability to blended learning mode among non-English majored Chinese learners in the College English course.

**Methods:**

The quantitative research method was employed in this study, and 595 respondents were eventually collected to enable the data analysis. The structural equation modeling (SEM) approach was used to analyze the data.

**Results:**

(1) The non-English majored learners’ adaptability to blended learning mode in College English was at a low level with a mean value of 2.26, indicating that students still suffer difficulties from class conducted in blended learning; (2) the fit indices were at the level of good fit, and it suggested the structural model had an overall good fit to the data as shown: *x*^2/^df = 2.496, RMESA = 0.050, GFI = 0.956, AGFI = 0.936, NFI = 0.968, RFI = 0.959, IFI = 0.980, TLI = 0.975, CFI = 0.980; (3) adaptability was positively related to contextual factors, self-efficacy, and motivation (*p* < 0.001); (4) contextual factors exerted an indirect effect on the adaptability not only through the separate mediating role of self-efficacy and motivation, but through the chain mediating role of self-efficacy and motivation (*p* < 0.01).

**Implications:**

This study theoretically extends previous studies on adaptability by investigating the affecting factors in the framework of social cognitive theory. More practically important is that this study sheds light on the impact mechanism of positive and enjoyable environment, self-efficacy and motivation on non-English majored learners’ adaptability to blended learning mode in English course, which would provide a vital insight for administrators and College English instructors to reconsider the role of learners’ responses in the mixed mode to improve their English achievement more effectively, as well as to enhance and promote their user experience of the offered blended learning service.

## Introduction

Information technologies are pervading the education landscape, and blended learning has emerged as an effective and efficient integration of traditional classroom learning with online learning (Neumeier, 2005; Graham, 2006), which evolved from cost and time effectiveness to the enhancement of flexible, active, and joyful learning experience of learners with different learning capabilities (Hussain et al., 2020; Malekigorji and Hatahet, 2020). Albeit its growing popularity in tertiary education (Norberg et al., 2011; Smith and Hill, 2019), blended learning as the “new normal” (Norberg et al., 2011) has not met learners’ expectations and achieved the desired remarkable effectiveness yet (Mirriahi et al., 2015; He, 2019), and many studies have been conducted to explore the constraints for the successful implementation of blended learning in different disciplines (Bansal, 2014; Tshabalala et al., 2014; Singh, 2021). Simultaneously, researchers and scholars have also been exploring a multitude of factors impeding its full potential in English teaching with their explicit focus on technology, attitude, pedagogical design, learning atmosphere and the personal factors of learners. For instance, Sharma and Barrett (2007) alluded to the language teachers’ uneasiness about technology use and learners’ insufficient accessibility to technological devices in a blended learning setting. Milthorpe et al. (2018) questioned the assumption of blended approach implementation, that is, all learners in blended English were unanimously tech-savvy and had no difficulty using technology. Other scholars suggested that the unsuccessful implementation of blended English courses was associated with the negative attitude of instructors and students. According to a case study made by Ja’ashan (2015), instructors were reluctant to blend English course due to a longer preparation time for the integration of both modes, and students were not committed to the mode due to concern over the poor internet connection, easy cheating, and teachers’ belated reply. Similarly, Liu and Yu (2012) noted that designing personalized and autonomous blended learning environment to engage students in the activities would be rather demanding on the English teachers’ pedagogical knowledge, whereas, Chen and Guo (2009) disclosed that some Chinese teachers had insufficient knowledge and practice in network-based teaching. Furthermore, the lack of a learning environment for intense collaboration and interaction to improve learners’ autonomous learning ability in blended setting was unveiled in previous studies (Maulan and Ibrahim, 2012; Zhang, 2013; Zhang and Song, 2015). In contrast, accumulating evidence indicated that students’ personal factors might impede their greater achievement in English, including low motivation, low self-efficacy, and inappropriate learning strategies in blended learning context (Meng, 2011; Liu and Yu, 2012; Ma and Jiang, 2013).

However, early studies seldom discussed students’ capacities to respond to the adoption of blended learning mode, or addressed the role of their adaptability in the transition to such a less traditional and student-centered autonomous learning mode within the blended, though it has been noted that changes and transitions the university students encountered would disrupt the routines and strongly affect their academic emotions as well as performance (Baggaley, 2013; Collie et al., 2017; Xiao et al., 2020). Unlike the traditional school environment in which students were taught and received knowledge passively, the blended learning environment encouraged students to construct knowledge actively (Ma and Jiang, 2013; Akbarov et al., 2018), and the change thereby caused the maladaptation of English learners in blended learning, such as having trouble critically thinking about the overloaded online information (Yang, 2009), overcoming negative emotions (Stockinger et al., 2021), and staying socially connected (Ja’ashan, 2015). Thus, the authors considered it necessary and pertinent to tackle the questions what the salient factors are and how they are affecting non-English majored learners’ adaptability to blended learning mode in English course. At the heart of the questions lie two research purposes:

1. Propose a conceptual model that explains the relations among affecting factors and learners’ adaptability to blended learning mode in College English course;
2. Test the conceptual model and examine the interrelationships among affecting factors and learners’ adaptability to blended learning mode in College English course.

## Literature Review and Hypothesis Development

### Adaptability

Adaptability was conceptualized by Martin et al. (2012) as an individual’s capacity to “constructively regulate psycho-behavioral functions in response to new, changing, and/or uncertain circumstances, conditions and situations” (p. 66). This construct involved one’s adjustment in cognition, behavior, and emotion (Martin et al., 2012, 2013), and was strongly related to positive outcomes at school and work (Collie and Martin, 2016). Indeed, a large body of studies has reached the consensus that students with higher adaptability would attain better learning outcomes, including higher satisfaction with the course (Holliman et al., 2018b), higher involvement in the academic activities (Collie et al., 2017; Holliman et al., 2018a), as well as higher level of course completion (Martin et al., 2013; Burns et al., 2017). With the prevalence of web-based learning, researchers showed concerns over learners’ adaptability in online education since it signified a sudden shift from traditional face-to-face mode to the framework of “self-regulated learning” (Zimmerman, 2002; Sheriston et al., 2019) and claimed for higher self-regulation, resilience, or buoyancy (Martin et al., 2013; Stockinger et al., 2021). For example, Zhang K. et al. (2020) investigated how Chinese university students responded to the adoption of the online learning format under COVID-19 and contended that adaptability was closely related to student engagement. They also disclosed the mediating role of academic emotion in the correlation. Likewise, Stockinger et al. (2021) studied 89 pre-service teachers taking online courses at South-German university after the outbreak of Covid-19 and showed that student adaptability was closely correlated to emotions and test scores. With respect to blended learning setting, Liao and Huang (2009) revealed that students’ adaptability in virtual communities positively affected their perceived ease of use and perceived usefulness of blended learning; Yang and Tong (2015) investigated Chinese students’ adaptation to the blended mode based on MOOC, and suggested that they were not well adapted to the mode. Taken together, existing research has shown the importance of learners’ adaptability in educational settings.

Given that adaptability would cause unique variance in individual psychological well-being and academic outcomes (Collie and Martin, 2016), researchers explored the affecting factors of student adaptability. Hartley and Bendixen (2001) discussed learners’ characteristics in their adaption to the internet age, incorporating their epistemological beliefs and self-regulatory skills. Cao et al. (2008) explained in theory that student adaptability in online learning was influenced by technology, learning environment and students’ meta-cognitive factors, namely, motivation, self-efficacy, and willpower. Wang and Zhou (2010) identified the factors influencing the adaptability of Chinese adult learners to online courses from dimensions of individual learning ability (learning strategy, attitude, learning skill, communication ability *via* network and ability to get help) and learning environment (usability, web environment, study environment, and changes in environment). Abbacan-Tuguic (2021) showed that students’ attitudes and readiness to use blended learning would affect adaptability in the blended environment. However, to our best knowledge, little empirical research has investigated factors affecting the adaptability of students to blended learning mode in English course, even though their problems in adaptation to a more constructivist mode (Rajkoomar and Raju, 2016) have been reported in numerous studies (Liu and Yu, 2012; Ma and Jiang, 2013; Ja’ashan, 2015; Tosun, 2015). Therefore, against the background of blended learning mode being widely adopted by universities in China to reform the College English teaching (Wang and Xu, 2015; Wang and Zhong, 2017), a study investigating the antecedents of adaptability among Chinese non-English majored learners would not only enrich body of adaptability literature, but provide unique and insightful evidence for promoting a mixed instruction mode rather than traditional teaching in Chinese higher education institutions.

### Social Cognitive Theory

Bandura’s social cognitive theory (1986) acknowledges “the social origins of human thought and action” (p. 12), and holds there exists a dynamic interaction “in which environmental events, personal factors, and behavior all operated as interacting determinants of each other” (p. 11). In learning specifically, personal factors refer to learner’s cognitive, emotional, and biological events (Maddux, 2013). Schunk (2001) defines environmental events as the “instructional environment” (p. 85), composed of social and physical factors, and behavior as the actions taken to achieve desired goals, such as “the instigation, direction, and persistence of achievement-related behaviors” (p. 83). According to this theory, learners are able to construct knowledge by taking the initiative to interact with the learning environment, instead of passively imitating direct experience (Bandura, 1986, 2001; Schunk, 2001; Schunk and DiBenedetto, 2021), which posits two critical assumptions: learning occurs in the interaction with the external environment (Prawat and Floden, 1994; Bandura, 2001; Schunk, 2001); and learners are agents capable of performing and adjusting behaviors to attain the given purposes (Bandura, 1986, 2001; Schunk and DiBenedetto, 2021). Thus, social cognitive theory lays a solid theoretical foundation for the blended learning mode that aims to encourage learners to build knowledge through self-paced autonomous learning and commitment to active learning (Tsai et al., 2011; Means et al., 2013). But in the meanwhile, learners are also challenged to adjust themselves to the changing format (Tsai et al., 2011). Therefore, social cognitive theory helps us to understand the environmental and personal factors affecting non-English majored learners’ adaptability to blended learning mode in College English.

### Hypotheses and Research Model

Based upon the aforementioned theoretical research, this study identified contextual environment (contextual factors), personal factors (self-efficacy and motivation) as the key determinants of a learner’s adaptability to blended learning mode in College English.

#### Contextual Factors

Researchers in education asserted the role of environment in learning and categorized it into “the physical and social environments in a classroom setting” (Wu et al., 2010, p. 157). Lam et al. (2012) synthesized the learning environment with instructional contexts and social relatedness contexts. To illustrate the online learning process, Khan (2005) developed an octagonal framework composed of pedagogy, technology, interface design, evaluation, management, resource support, ethics, and institutions. The present study addressed adaptability in the blended learning environment and defined contextual factors as “the non-learner characteristics that constitute the learning environment that facilitates students’ learning activities in the blended learning context” (p. 52), and tied it to “institutional adoption, teaching competence, technology quality, course content and blended learning community culture” (Yang et al., 2021, p. 53).

Maddux (2013) once commented that most research in scientific psychology was to study how people responded and adjusted themselves to the changing environment, and the impact of learning environment on adaptability could be found in ample research. Bonk and Graham (2012) indicated that learners’ difficulty in adjusting to the blended learning was partly explained by technology, course design, peer interaction, as well as the integration of the teaching modes. Xu and Jaggars (2013) found that subject areas were related to online learners’ adaptability. In a recent study, Hatlevik and Bjarnø (2021) viewed digital distractions as a major obstacle to a student’s learning behavior, showing t to what extent the students were able to cope with the distractions that affected their studying approaches. Based on previous studies, we proposed the following hypothesis:

**H**_1_. Contextual factors (CF) will have a positive and direct effect on non-English majored learners’ adaptability (AD) to blended learning mode in College English.

#### Personal Factors

Social cognitive theory viewed students as active seekers of knowledge who were able to exercise control over learning activities and outcomes (Bandura, 1986; Schunk, 2001). Of the personal factors were two major factors closely related to the self-regulated learning format, namely, self-efficacy and motivation (Zimmerman and Martinez-Pons, 1992; Schunk, 2001; Kuo et al., 2014; Hatlevik and Bjarnø, 2021). Bandura (1986) proposed that any psychological or behavioral change should be traced back to one’s sense of self-efficacy, which refers to “people’s beliefs about their capabilities to exercise control over events that affect their lives” (Bandura and Wood, 1989, p. 1175). Moreover, Maddux (2013) contended that one’s belief about abilities to cope with and control the changes was critical to their adaptation and adjustment. Previous studies have shown the impact of self-efficacy on adaptability at school or the workplace (Cao et al., 2008; Wang and Zhou, 2010; Collie and Martin, 2016). One example was that Chemers et al. (2001) conducted a longitudinal study among the freshmen and found that their academic self-efficacy positively affected their personal adjustment in university life. No study has enunciated the impact of self-efficacy on students’ adaptability to blended learning mode in College English, though existing literature about its critical role in technology-based learning (Shea and Bidjerano, 2010; Wu et al., 2010; Hatlevik and Bjarnø, 2021) and in English learning (Gahunga, 2009; Yang et al., 2013) led us to consider the relationship between students’ belief in their abilities to learn English in the blended learning environment and their adaptability. For instance, Hatlevik and Bjarnø (2021) found that students’ self-efficacy on information technology positively affected their learning strategy use and willingness to spend time on the learning tasks in a digital-technology based learning environment. Gahunga (2009) and Yang et al. (2013) found that English learners’ high level of self-efficacy would promote their English ability. Accordingly, we made the following hypothesis:

**H**_2_. Non-English majored learners’ self-efficacy (SE) will be positively related to their adaptability (AD) to blended learning mode in College English.

Motivation generally denotes the reasons driving people to act and sustain behavior (Oxford and Shearin, 1994; Schunk and DiBenedetto, 2021), and researchers distinguish intrinsic motivation from extrinsic motivation (Dörnyei, 2001; Ryan et al., 2010). According to Dörnyei (2001). Intrinsic motivation refers to the motivation arising from the inner rewards within the individual, such as pure enjoyment or interest, whereas, extrinsic motivation involves engagement in behavior to gain external rewards, such as prize and money, or to avoid punishment. In English learning, intrinsic motivation was more about learners’ identification with the target culture and people, while extrinsic motivation reflects the learners’ aim to gain high grade and to get a job (Liu and Yu, 2012); and language learning is never motivated by a single type (Alizadeh, 2016).

McClelland (1985) proposed that motivation was one of the key determinants of achievement-related behavior, and Dörnyei (2001) also indicated that attainment in language learning was partly determined by motivation. Previous studies have shown that in the blended learning environment, English learning motivation was intrinsically related to their autonomy (Banditvilai, 2016), choice of learning strategies (Liu and Yu, 2012) as well as language skills (Adas and Bakir, 2013; Tosun, 2015). Furthermore, in a changing situation, one’s motivation to master changes and achieve goals contributed to effective adaptation (Maddux, 2013), and many empirical studies have been conducted to affirm the positive relationship between learning motivation and adaptability to a new environment (Sheriston et al., 2019; Azizi et al., 2020; Abbacan-Tuguic, 2021). We therefore proposed the following hypothesis:

**H**_3_. Non-English majored learners’ motivation (MO) will be positively related to their adaptability (AD) to blended learning mode in College English.

The triadic model of social cognitive theory showed that self-efficacy and motivation in the context of learning could be influenced by environmental factors such as teachers, peers, and school climate (Bandura, 1986; Schunk, 2001; Maddux, 2013; Schunk and DiBenedetto, 2021). Observing successful models, setting difficult but attainable goals, making comparisons with others, and receiving performance feedback and rewards were taken as effective instructional and social means to enhance students’ self-efficacy and motivation (Schunk, 2001; Maddux, 2013; Schunk and DiBenedetto, 2021). Besides, studies in English course with the blended learning mode showed that improvement of technology quality (Mayadas, 1998; Tosun, 2015), instructors’ design of blended courses (Yoon and Lee, 2010; Banditvilai, 2016), peer interaction (Yoon and Lee, 2010; Banditvilai, 2016), and institutional support (Sharma and Barrett, 2007) contributed to learners’ self-efficacy and motivation. Accordingly, we made the following hypotheses:

**H**_4_. Contextual factors (CF) will be positively related to non-English majored learners’ self-efficacy (SE) in College English with blended learning mode.
**H**_5_. Contextual factors (CF) will be positively related to non-English majored learners’ motivation (MO) in College English with blended learning mode.

#### Personal Factors as Mediators

The literature reviewed above support our hypotheses on the direct effect of contextual factors on personal factors (self-efficacy and motivation) and adaptability. However, we considered that contextual factors might also influence adaptability indirectly through self-efficacy and motivation. Försterling (1986) once proposed that the correlation between external events and behavior would be mediated by self-efficacy, and Bandura (1986) showed that adaptive behaviors was affected by models through the mediation of changes in self-efficacy. In the blended learning environment, learners’ self-efficacy was found to take up the mediating role between the learning environment and their behavior. For example, Shea and Bidjerano (2010) surveyed the online students and found that self-efficacy was mediating the influence of physical online environment and social supportive environment on their self-regulation behavior. Chu and Tsai (2009) showed that the effect of internet usage on adults’ preference of online courses was mediated by their internet self-efficacy.

There does not appear to be any study on the mediating role of motivation between blended learning environment and students’ adaptability, though previous studies have shown that motivation was most susceptible to environmental factors (Iwaniec, 2018) and closely related to adaptive behaviors in novel situations. For instance, Ryan and Patrick (2001) indicated that an environment of mutual respect increased students’ motivation, leading to a lower level of anxiety and worries. Besides, the mediating role of motivation between technology characteristics and e-learners’ behaviors after training was explicitly shown in the research of Bukhari et al. (2014).

In summary, the direct effects and indirect effects among the variables in the reviewed works indicated that self-efficacy and motivation might mediate the relationship between contextual factors and adaptability in the blended learning environment. Hence, we proposed the following hypotheses:

**H**_6_. Non-English majored learners’ self-efficacy (SE) will mediate the relationship between contextual factors (CF) and adaptability (AD) to blended learning mode in College English.
**H**_7_. Non-English majored learners’ motivation (MO) will mediate the relationship between contextual factors (CF) and adaptability (AD) to blended learning mode in College English.

In addition to the one-path indirect effect, contextual factors might have an indirect effect on learners’ adaptability through the chain mediating role of self-efficacy and motivation owing to their positive association. Social cognitive theory postulated that one’s belief in abilities to cope with challenges and make progress to achieve the desired outcomes was apt to generate and sustain motivation (Bandura and Schunk, 1981; Schunk, 2001). Regarding self-regulated learning, Zimmernan and Schunk (2001) indicated that self-efficacy affected students’ motivation of activity choice, effort and persistence in self-regulation. Liang and Wu (2010) showed that higher level of internet self-efficacy would lead to higher motivation to use web-based learning; and Wang (2008) suggested that non-English majors with high self-efficacy were more motivated in face of obstacles and challenges. Accordingly, we proposed the following hypothesis:

**H**_8_. Self-efficacy (SE) and motivation (MO) will have mediating effect in the relationship between contextual factors (CF) and non-English majored learners’ adaptability (AD) in College English with blended learning mode.

**FIGURE 1:**
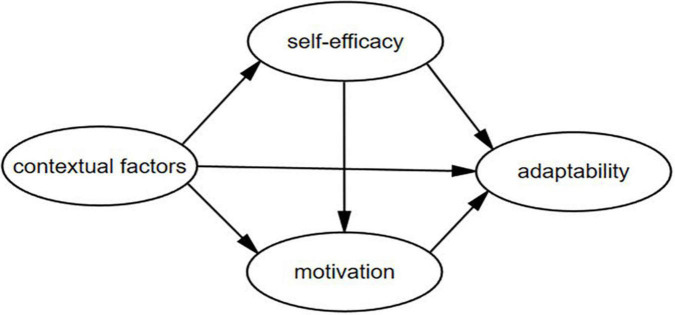
The research model for adaptability in blended learning environment.

The complete research model is presented in Figure 1.

## Materials and Methods

### Sample and Sampling Method

This cross-sectional study was conducted at the universities administered by provincial governments in Sichuan province, China.

In the present study, the target population was the juniors and sophomores in the first semester of 2021–2022 who were taking College English with the blended learning mode. The sample size was originated from the sample size table of Krejcie and Morgan (1970), in which 384 samples were required with a confidence level of 95% and a margin error of 5%. Meanwhile, a rate of 10–20% for non-respondents should be considered in random sampling (Donald, 1967; Miller and Smith, 1983). Therefore, the minimum sample size was 422.

Multi-stage random sampling (Onwuegbuzie and Leech, 2007) was applied to select the samples. The universities in Sichuan province were firstly stratified and 5 universities were then selected to represent different classes (1 medical school out of 4, 1 normal university out of 5, 1 independent institute out of 8, and 2 comprehensive universities out of 15). Then the students at these schools were screened by stratifying them with the condition “admitted in September 2019 or 2020,” “majored in liberal arts or sciences” but excluding those majored in “English, Fine Arts, Music and Sports.” The screened students from each school were numbered and then selected from the 2 grades and different departments at random. 900 students were selected and an online questionnaire link was sent to the administrators or College English teachers from the 5 schools who accepted our request to help collect the data. All of the participants were clearly informed that the questionnaire was anonymous and 10–15 min would be given. A total of 819 non-English majored learners completed the online questionnaire and the response rate was 91%. 224 were not included in the analysis because either all of the items were marked with the same answer or the time on the completion was less than 3 min. Ultimately, 595 questionnaires were finally analyzed, which exceeded the minimum sample size.

### Instrument

To achieve the research objectives of the present study, the researchers used a two-part structured questionnaire. The first part was dedicated to participants’ demographic profile, including gender, major, grade, prior experience in network learning mode before coming to college, and the percentage of online learning replacing face-to-face learning in College English with the blended learning mode. The second part included 15 questions on a 5-point Lickert scale, intended to measure the 4 constructs, namely contextual factors (CF), self-efficacy (SE), motivation (MO), and adaptability (AD).

Since some of the items in the questionnaire were originally in English, the researchers translated them into Chinese first. To establish the content validity of the questionnaire, the translated questionnaire was proofread by 4 College English teachers in terms of clarity, language accuracy, and verbal structure, and the 5 non-English majored learners were invited to find whether they had difficulty understanding the instructions and questions in the questionnaire. Moreover, 4 professors in the English department from different schools were consulted. A pilot study on 80 randomly selected non-English majored learners at the university where the author was working and the reliability of the questionnaire were assessed. It should be mentioned that these students only participated in the primary survey. Based on the results of the primary investigation and the comment of the professors, the questionnaire was improved before being distributed to the participants.

The construct of contextual factors was related to institutional adoption, teaching competence, technology quality, course content, and the blended learning community culture (Yang et al., 2021). This construct was measured with 5 items from the 5-point Likert scale used by Yang and Tong (2015). No reliability was reported in their research, but the means in the dimensions of school, teacher, technology, course content, and emotional support were higher than 3.5. We revised the original statements with the situation of College English in the blended learning environment. For instance, we modified the statement on technology based on Technology Acceptance Model (Davis, 1989), and it was like “At our school, the learning management system and Apps for College English course with blended mode were useful.” Reliability (Cronbach’s alpha) of the revised scale was 0.935.

Self-efficacy was measured with the Online Learning Value and Self-Efficacy Scale (OLVSES), which was developed by Artino and McCoach (2008) for self-paced online learning and composed of 5 items (Cronbach’s alpha = 0.87). All the five items were modified and translated into Chinese, and the Cronbach’s alpha reached 0.881. However, the principle component analysis later suggested that 2 items should be excluded and the reliability of the 3 items was 0.86. The value was higher than 0.8, indicating a good internal consistency (Schweizer, 2011). Moreover, the items in this study were closely related to non-English majored learners’ belief in their capability of independent online learning and performance in English under the blended setting. For example, the scale contained items like “I am confident I can learn without the presence of an English instructor to assist me.”

Motivation was measured with the items used by Peng and Fu (2021) when they studied the impact of Chinese English learners’ learning motivation on English achievement within the blended setting. The items they used synergized learners’ intrinsic motivation and extrinsic motivation, which was in line with most scholars’ viewpoints (Bernaus and Gardner, 2008; Ryan et al., 2010). The Cronbach’s alpha of the scale was 0.799. In this study, the scale consisted of 3 items and the Cronbach’s alpha was 0.755, which reached the acceptable reliability degree of 0.70 (Schweizer, 2011).

The adaptability scale developed by Martin et al. (2012) was utilized to measure adaptability. Martin et al. (2012) suggested that a single global factor be used due to the strong interrelatedness between cognitive-behavioral adaptability and emotional adaptability. Besides, the reliability and validity of the scale has been further verified in previous research (Burns et al., 2017; Holliman et al., 2018b; Stockinger et al., 2021). This scale was therefore revised for the present study to assess non-English majored learners’ adaptability in College English with blended learning mode with items like “I am able to revise the way I think about College English with blended learning mode to help me through it.” and “To help me adapt to College English with blended learning mode, I am able to draw on positive feelings and emotions (e.g., enjoyment and satisfaction).” In the current study, the Cronbach’s alpha value of 0.910 indicated their excellent internal consistency.

### Data Analysis

We performed data analysis using SPSS (IBM SPSS 25.0, SPSS Inc., Armonk, NY, United States) and AMOS version 24. First, we conducted frequency analysis, reliability analysis, and correlation analysis with SPSS. Next, we tested the structure of the model by conducting confirmatory factor analysis (CFA) with Maximum Likelihood Estimation in AMOS. Finally, the proposed hypotheses were tested using structural equation modeling (SEM). Bias-corrected bootstrap was run to examine the mediating effect of multiple mediating variables (Preacher and Hayes, 2008). We used 1,000 subsamples and set a 95% confidence level for the significance of a*b in the multiple mediator models.

## Results

### Demographic Information

Among the 595 participants 263 were men (44.2%) and 332 women (55.8%). Out of the total 188 (31.6%) were majored in liberal arts and 407 (68.4%) were in science. Overall, 232 students (38.99%) reported that they had the experience of online learning or blended learning before coming to college, whereas 363 (61.01%) did not have. In terms of the percentage of online learning replacing face-to-face learning, 40 students (7%) reported that there was no reduction of face-to-face learning time in College English class, 383 (64%) reported the percentage of online learning session was below 50%, and 172 (29%) reported over 50% of online learning session (Table 1).

**TABLE 1:** Demographic information of participants.

Characteristics	Items	Numbers (%)
Gender	Male	263 (44.2%)
	Female	332 (55.8%)
Grade	Sophomore	232 (39%)
	Junior	363 (61%)
Major	Liberal arts	188 (31.6%)
	Science	407 (68.4%)
Network learning experience before coming to college	Yes	232 (38.99%)
	No	363 (61.01%)
Percentage of online learning in College English course	0	40 (7%)
	<50%	383 (64%)
	>50%	172 (29%)

*N = 595.*

### Correlation Analysis for the Confirmatory Factor Analysis Measurement Model

Pearson correlation analysis was executed to determine the relations between the variables, and the strength was based on the correlation coefficient (*r*-value). As displayed in Table 2, all the variables were positively related. Specifically, contextual factors had a significant positive correlation with the other three variables, SE (*r* = 0.671; *p* < 0.01), MO (*r* = 0.589; *p* < 0.01), and AD (*r* = 0.662; *p* < 0.01). Students with a high level of self-efficacy also had strong motivation (*r* = 0.508; *p* < 0.01) and strong adaptability (*r* = 0.645; *p* < 0.01). Besides, a positive correlation existed between motivation and adaptability (*r* = 0.621; *p* < 0.01). The coefficient values between the variables suggested that both contextual factors and self-efficacy had a moderate correlation with motivation (0.5 < *r* < 0.6, and *p* < 0.05), whereas other correlations between the variables were strong (*r* > 0.60, and *p* < 0.05).

**TABLE 2:** Pearson correlations of the variables.

Index	*M*	*SD*	1	2	3	4
CF	2.05	0.67	–			
SE	2.36	0.80	0.67**	–		
MO	2.15	0.68	0.59**	0.51**	–	
AD	2.26	0.68	0.66**	0.65**	0.62**	–

N = 595. CF, contextual factors; SE, self-efficacy; MO, motivation; AD, adaptability. **p < 0.01.

### Confirmatory Factor Analysis of Measurement Model

Cronbach’s alpha, factor loadings, composite reliability (CR), and average variance extracted (AVE) should be analyzed to verify the suitableness of the measurement model (Wu, 2010). As presented in Table 3, the standardized factor loading of each item was above the threshold value of 0.5 (Carmines and Zeller, 1979), indicating that the items could reflect the latent variables accurately. In this study, the Cronbach’s alpha values for the scales were from 0.76 to 0.935, and composite reliability values were higher than 0.75. Furthermore, the values of average variance extracted were above the cut-off value of 0.50, indicating that the modified measurement model had good convergent validity.

**TABLE 3:** Construct reliability, factor loadings, composite reliability, and average variance extracted.

Constructs	Items	Cronbach’s alpha ≥ 0.7	Factor loadings > 0.5	CR ≥ 0.7	AVE ≥ 0.5
CF	CF1		0.744		
	CF2		0.804		
	CF3	0.935	0.852	0.914	0.681
	CF4		0.868		
	CF5		0.851		
SE	SE1		0.748		
	SE2	0.88	0.857	0.860	0.672
	SE3		0.850		
MO	MO1		0.771		
	MO2	0.76	0.764	0.796	0.565
	MO3		0.718		
AD	AD1		0.860		
	AD2	0.91	0.896	0.912	0.721
	AD3		0.849		
	AD4		0.788		

*N = 595. CF, contextual factors; SE, self-efficacy; MO, motivation; AD, adaptability.*

Another important evidence of construct validity was discriminant validity, which supposed that the measured constructs should not be highly correlated to each other (Henseler et al., 2015). The discriminant validity could be determined by judging whether the square root of AVE of the particular construct was greater than its correlation with other constructs (Wu, 2010). The square root of AVE presented in parentheses diagonally of Table 4 showed that the questionnaire for this study had desirable discriminant validity.

**TABLE 4:** Discriminant validity.

Variables	1	2	3	4
CF	**0.825**			
SE	0.767***	**0.820**		
MO	0.680***	0.621***	**0.752**	
AD	0.718	0.732	0.695	**0.849**

N = 595, square root of AVE was in bold and presented in parentheses diagonally. ***p < 0.001.

### Structural Model Test and Hypotheses Testing

Since the CFA analysis results suggested that the measurement model was acceptable, structural equation modeling (SEM) was run in AMOS to test the goodness-of-fit on the structural model. The fit indices in Table 5 showed that though the ratio of chi-square to degrees of freedom (*x*^2/^df = 2.496) was higher than that of the good-fit level (0 ≤ *x*^2/^df ≤ 2), adjusted goodness of fit index (AGFI) was lower than 0.95, and most fit indices were above the level of good fit (Schermelleh-Engel et al., 2003; Wu, 2010). The fit indices therefore suggested that the modified structural model had an overall good fit to the data.

**TABLE 5:** Model fit index.

Fit index	*x*^2^/df	RMSEA	SRMR	GFI	AGFI	NFI	RFI	IFI	TLI	CFI
Proposed value	≤2	<0.05	<0.05	≥0.95	≥0.95	≥0.95	≥0.95	≥0.95	≥0.95	≥0.95
Estimated value	2.496	0.050	0.317	0.956	0.936	0.968	0.959	0.980	0.975	0.980

**FIGURE 2:**
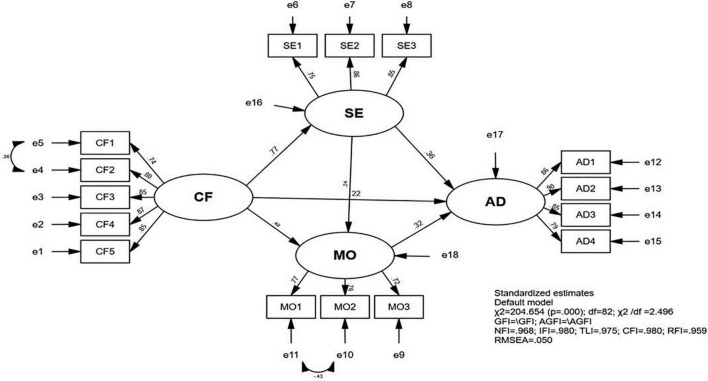
The result of path coefficients of the final model.

Additionally, the hypotheses on the relationships of the latent constructs were tested through SEM. Figure 2 and Table 6 showed the final SEM model and the estimated standardized direct effects for each hypothesized relationship (H1–H5). The critical ratio value was greater than 2.58 (Biswas et al., 2006) and the *p*-value for each path was significant at the 0.001 level (two-tailed), indicating that the 6 hypotheses we posited on the relationships among the variables were all accepted.

**TABLE 6:** Results of structural model testing.

	Path	Path coefficient	S.E.	C.R.
H1	CF → AD	0.224***	0.063	3.724
H2	SE → AD	0.364***	0.057	6.262
H3	MO → AD	0.316***	0.055	6.334
H4	CF → SE	0.767***	0.050	16.241
H5	CF → MO	0.494***	0.067	7.025

*CF, contextual factors; SE, self-efficacy; MO, motivation; AD, adaptability. S.E., standard error; C.R., critical ratio.*

***p < 0.001.

As shown in Figure 2, CF could exert an indirect effect on AD through three paths. To test the indirect effect, bootstrap analysis was conducted (Table 7). The standardized indirect effect of CF on AD through SE was 0.277, and the 95% CI (0.2011–0.445) did not contain 0, suggesting the indirect effect was statistically significant. The path from CF to AD through MO was also significant, and the standardized indirect mediating effect was 0.157. The 95% CI (0.143, 0.449) did not contain 0. Furthermore, the chain-mediating effect of SE and MO between CF and AD was 0.059. H6, H7, and H8 on the indirect effects of the variables were therefore supported.

**TABLE 7:** Bootstrap analysis of mediating effect significance test for the final model.

	Model path	Standardized indirect effect	*p*	BootLLCI (95% CI)	BootULCI (95% CI)
H6	CF → SE → AD	0.277**	0.002	0.201	0.445
H7	CF → MO → AD	0.157**	0.002	0.143	0.449
H8	CF → SE → MO → AD	0.059**	0.007	0.030	0.090

*CF, contextual factors; SE, self-efficacy; MO, motivation; AD, adaptability.*

**p < 0.01.

## Discussion and Implications

### Discussion on the Significant Results

Blended learning has been widely adopted in tertiary education to improve the teaching and learning quality. But only when this system provided its end users (students) with a pleasant and enjoyable user experience, it could achieve the desired effectiveness (Raaij and Schepers, 2008). Student adaptability as a relatively new construct on learner’s behavioral-cognitive and emotional response to uncertainty and novelty should not be ignored in the process of blended learning implementation. Our study, therefore, aimed to investigate the relationships between contextual factors, self-efficacy, motivation, and adaptability of the non-English majored learners’ to blended learning mode in College English. The results showed that contextual factors of the blended learning directed and positively affected students’ individual attributes (self-efficacy and motivation), and adaptability in the novel learning format. With respect to the impact on adaptability, contextual factors, self-efficacy, and motivation were all positive predictors of it in a significant way. In addition, contextual factors indirectly affected adaptability through self-efficacy and motivation.

Our research results echoed previous studies that asserted the critical role of students’ adaptability. It was considered to positively predict students’ achievement at school and satisfaction in life (Burns et al., 2017; Collie et al., 2017; Holliman et al., 2018a; Sheriston et al., 2019). Zhang K. et al. (2020) and Stockinger et al. (2021) further reported that adaptability was strongly associated with students’ emotional wellbeing and academic success in the transition to online learning due to the sudden outbreak of COVID-19. However, in the context of technology-based learning, learners were found to have trouble adapting to the student-centered and self-paced learning mode, either in online learning or blended learning (Yang, 2009; Tsai et al., 2011; Yang and Tong, 2015; Akbarov et al., 2018). The present research underpinned the existing research with the evidence that the score for adaptability was not high (mean = 2.26), indicating that students were not well adapted to the blended learning mode of College English, though it has been applied in tertiary education for years. The school administrators and College English teachers should be concerned about this result since adaptability was strongly related to students’ positive academic outcomes and satisfaction of blended learning service quality (Abu Seman et al., 2018). Despite the low level of adaptability,

the empirical results showed that it might be improved through contextual factors, student’s self-efficacy, and motivation since it was significantly impacted by them all in the present study. This finding was completely consistent with the theoretical foundation of blended learning and social cognitive theory. According to this theory, learning was not acquisition, but construction through learners’ interaction with the environment and behaviors would be strongly stimulated by environmental and personal factors (Bandura, 1986; Puil et al., 2019), and thus a thoughtful integration of information communication technology in face-to-face teaching and enhancement of learners’ self-efficacy and motivation would increase their adaptability and active participation in the activities (Tsai et al., 2011; Rajkoomar and Raju, 2016). Many scholars, therefore, committed to blended learning environment design to facilitate students’ learning (Sharma and Barrett, 2007; Tsai et al., 2011; Huang and Li, 2014). Sarıtepeci and Çakır (2015) and Elhoseny et al. (2017) especially highlighted that the blended learning environment makes students adaptable so that they can prioritize their personal and inner emotional needs. Though few studies quantitatively assessed the impact of contextual factors on adaptability, our study filled the gap by showing that contextual factors were positively related to adaptability with a direct effect of 0.224. In contrast, self-efficacy had the largest direct effect size on adaptability with a direct effect of 0.36 in our study. This finding was theoretically defensible because blended learning, being student-centered, was cognitively and metacognitively demanding for students (Bandura, 2001; Zimmerman, 2002; Pintrich, 2004), who should take the responsibility of knowledge construction by using various intentional strategies (Pintrich, 2004; Tsai et al., 2011). Without the physical presence of teachers, a learner’s self-efficacy would be the key factor for academic success in the self-regulated learning environment (Tsai et al., 2011; Korr et al., 2012). In the English learning domain, numerous empirical studies have reached the consensus that self-efficacy was positively related to learning strategies, motivation, engagement, satisfaction, and academic outcomes (Liu and Yu, 2012; Alsowat, 2016; Hu et al., 2021). Noticeably, Pappamihiel (2002) argued that students with a lower level of self-efficacy were more likely to suffer from anxiety in English learning, which further supported our position that self-efficacy would affect adaptability involving cognitive, behavioral, and emotional adjustment (Martin et al., 2012). Our hypothesis on the positive correlation between motivation and adaptability was also accepted. This result could be explained by previous research, which confirmed that motivation had a positive relation with students’ performance in English learning. For instance, Bernaus and Gardner (2008) showed that students English achievement was significantly affected by their integrative and instrumental motivation. Likewise, Peng and Fu (2021) found that Chinese students’ motivation, especially intrinsic motivation, was closely related to their English linguistic competence in a blended learning environment. Besides, several studies investigated the effect of motivation on students’ adaptability, such as Adekola et al. (2017) indicated that motivation would influence learners’ learning strategies to handle the problems in blended learning, and Wang and Zhou (2010) demonstrated that adults’ extrinsic motivation was positively related to their adaptability in online learning. Together, our hypotheses on the direct effect of the variables on adaptability were all confirmed in the present study.

In addition to the direct effect, our study also disclosed that contextual factors affected adaptability significantly through the mediation role of self-efficacy and motivation due to their inherent correlation. Previous studies have clarified that learners’ self-efficacy and motivation were significantly impacted by contextual factors in dimensions like teacher, technology, interaction, etc. (Liu and Yu, 2012; Moskovsky et al., 2013; Yang et al., 2013; Kuo et al., 2014; Peng and Fu, 2021). With respect to the mediating effect, Wang (2011) indicated that teaching strategies would promote students’ intrinsic motivation in English writing through self-efficacy. Tian et al. (2018) investigated how self-efficacy and intrinsic motivation partly mediated the effect of students’ mathematics metacognitive knowledge on their performance. In line with existing research, contextual factors of blended learning exerted a significant indirect effect on adaptability through three paths, two paths with separate mediating variables and one path with chain mediating variables. Of these, the indirect effect through self-efficacy (0.277) was stronger than that through motivation (0.157). This result could be explained by that students were at a low level of self-efficacy in technology-based learning environment (Yang and Tong, 2015), which held true in our study, with a mean of 2.36 for self-efficacy, and those students who were used to the face-to-face learning format in K-12 education were less dependent and insufficient in autonomous learning strategies, and needed more guidance and supervision from the external environment (Tsai et al., 2011).

To sum up, the positive relationships among the variables notwithstanding, the low level of each construct detected in our study (CF = 2.05, SE = 2.36, MO = 2.05, AD = 2.26) suggested that the current blended learning service was not favorable enough for many non-English majored learners, without lowering negative emotional mood (anxiety, frustration, and boredom), increasing motivation or promoting involvement in blended learning activities, which was in line with the findings of a previous research (Besser et al., 2020; Zhang Z. et al., 2020). Consequently, a positive and enjoyable blended learning environment should be emphasized in universities to enable learners to feel mastery of English learning with the novel mode; foster interest in the language; reduce negative feelings like anxiety, helplessness, or boredom, and therefore go through the transition from traditional face-to-face learning to blended learning successfully.

### Implications

Theoretically, to our best knowledge, this study was the first to investigate the predicting factors of student adaptability to blended learning mode and their interrelationships utilizing structural equation modeling. This attempt furthers the existing research mainly focusing on adaptability as positive predictor of learning outcomes. Specifically, the present study grounded in social cognitive theory highlights that changes in both learning environment and personal attributes may induce students’ adaptive responses.

Our study had practical implications for English teaching in the blended learning setting. It asserts the active force of English students’ cognitive-behavioral and emotional adaptation in the changing situation and might help explain why blended learning is yet to achieve the desired effectiveness since previous studies noticed that students had trouble adapting to the novel blended format for lacking abilities to manage time, deal with negative emotions, maintain motivation, and apply proper learning strategies (Tsai et al., 2011; Ma and Jiang, 2013; Yang and Tong, 2015). The findings in the direct and indirect effects of the affecting factors on non-English majored learners’ adaptability in blended learning were significant, for they explained in-depth the process of how adaptability could be affected by contextual factors and personal factors like self-efficacy and motivation. As a result, this study might be insightful for blended learning designers and practitioners to take into account learners’ emotional needs and explore means to enhance their adaptability, which certainly leads to students’ higher user satisfaction with blended learning service quality (Abu Seman et al., 2018).

## Conclusion

Theoretically based on social cognitive theory, this study proposed a model explaining the relations among contextual factors (environment), self-efficacy, motivation (personal factors), and adaptability (behavior) in a blended learning setting. The relationships among the variables were tested in a population of non-English majored learners of College English. In general, the results showed that the participants did not have a high level of adaptability in the blended learning setting, which was different from the traditional face-to-face instruction mode. However, the study revealed that adaptability could be promoted by contextual factors, self-efficacy and motivation since they exerted a positive and direct impact on adaptability. In addition to the direct effect, the findings also indicated that there existed several paths that adaptability could be indirectly affected due to the mediating role of self-efficacy and motivation. These findings are beneficial to the stakeholders who are adopting or attempting to adopt the blended leaning mode in English teaching to consider the role of students’ adaptability, which is pivotal for both their academic emotions and learning outcomes.

Several limitations need to be addressed when evaluating the current study. First, despite our attempt to generalize our findings by stratifying the universities in different classes, the participants were selected from the provincial administered universities in Sichuan province. Therefore it might be possible that different findings would be generated in the population of universities either in different regions or those under central ministries and agencies. Second, the model was only examined in the course of College English, and future research was therefore suggested to expand into other courses to verify the results. Third, the demographical variables were not considered in our study, which might lead to statistical bias; thus, future research might conduct a variance analysis for different groups to enhance the statistical power, such as gender, major, and experience in technology-based learning.

## Data Availability Statement

The raw data supporting the conclusions of this article will be made available by the authors, without undue reservation.

## Ethics Statement

The studies involving human participants were reviewed and approved by Panzhihua University, China. The patients/participants provided their written informed consent to participate in this study.

## Author Contributions

SY was responsible for the data collection, data analysis, and manuscript writing. RP translated and modified the questionnaire and proofread and modified the first draft of the manuscript. Both authors contributed to the design of the study and approved the submitted version.

## Conflict of Interest

The authors declare that the research was conducted in the absence of any commercial or financial relationships that could be construed as a potential conflict of interest.

## Publisher’s Note

All claims expressed in this article are solely those of the authors and do not necessarily represent those of their affiliated organizations, or those of the publisher, the editors and the reviewers. Any product that may be evaluated in this article, or claim that may be made by its manufacturer, is not guaranteed or endorsed by the publisher.
